# Self-perceived health of patients hospitalized due to non-psychiatric conditions: associations with psychiatric comorbidities and substance use

**DOI:** 10.1192/j.eurpsy.2024.1024

**Published:** 2024-08-27

**Authors:** M. Pons-Cabrera, E. Caballería-Lamora, L. Navarro-Cortés, M. Balcells-Oliveró, L. Pintor-Pérez, H. López-Pelayo

**Affiliations:** ^1^Health and Addictions Research Group (Grup de Recerca Emergent, 2021 SGR 01158, AGAUR). IDIBAPS. Addictions Unit. Psychiatry and Psychology Service. ICN; ^2^Consultation-Liaison Psychiatry Unit. Psychiatry and Psychology Service. ICN, Hospital Clínic de Barcelona, Barcelona, Spain

## Abstract

**Introduction:**

Self-perceived health (SPH) is an epidemiologically used variable, recognized as a subjective yet predictive indicator of mortality (Bopp *et al*. Plos One 2012; 7:e30795) SPH, among other subjective indicators, such as quality of life, contributes to understanding an individual’s overall experience and well-being. While health information, including medical diagnoses given by physicians, forms a substantial part of an individual’s subjective health (Falconer & Quesnel-Vallée, 2017; 190 227-236) the World Health Organization (WHO, 2014) defines health not only by the absence of somatic diseases but also encompasses components of social and mental well-being.

**Objectives:**

This study aims to explore factors associated with a poorer level of self-perceived health in inpatients due to non-psychiatric conditions with a focus on mental health and substance use-related factors.

**Methods:**

We recruited 800 patients during their hospital stay for various pathologies in cardiology, pneumology, internal medicine, and gastroenterology units. Self-reported sociodemographic variables and well-being-related variables, such as SPH, were collected during admission. The MINI Neuropsychiatric Interview was administered to screen for psychiatric conditions, the ASSIST scale assessed the risk related to the use of various substances. Data on the frequency and quantity of substance use, in the three months prior to admission, were also recorded by timeline follow-back. Information on the severity of somatic comorbidity was gathered using the Charlson Comorbidity Index. Non-parametric tests compared SPH in different groups, and variables showing significant differences were included in a multivariate linear regression analysis. This study obtained approval from the ethics committee.

**Results:**

Significant and clinically relevant differences were found in the SPH of patients with anxiety disorders, depressive disorders, and bipolar disorders. These patients reported lower SPH than those without these comorbidities. Patients scoring medium or high risk on the ASSIST scale for tobacco, alcohol, and cannabis also demonstrated lower SPH compared to those with low-risk scores. In the multivariate analysis, accounting for confounding factors, including comorbidity severity, patients with depressive disorders maintained statistically significant lower levels of SPH (ß = -13.391, p < 0.001), as did those with bipolar disorders (ß = -6.096, p = 0.019).

**Conclusions:**

Patients with anxiety, depressive, or bipolar disorders, as well as those with higher-risk use of tobacco, alcohol, and cannabis, exhibited lower SPH. After adjusting for other relevant factors, such as diagnosed somatic pathology, patients with affective disorders continued to score lower in SPH levels. Proper attention and management of psychiatric comorbidities and substance use are crucial in medical hospital settings.

**Disclosure of Interest:**

M. Pons-Cabrera Employee of: This work has been funded by Contractes Clínic de Recerca “Emili Letang - Josep Font” 2021 granted by Hospital Clínic of Barcelona, E. Caballería-Lamora: None Declared, L. Navarro-Cortés: None Declared, M. Balcells-Oliveró: None Declared, L. Pintor-Pérez: None Declared, H. López-Pelayo: None Declared

